# Clinical cases: asthma and obesity

**DOI:** 10.1186/2045-7022-5-S2-P11

**Published:** 2015-03-23

**Authors:** KS Ryabova, LA Goryachkina, DS Fomina

**Affiliations:** 1 Russian Medical Academy of Postgraduate Education, Department of allergology and immunology, Moscow, Russia; 2 Russian Medical Academy of Postgraduate Education, Department of allergology and immunology, Moscow, Russia

## Background

Recently published issues on asthma phenotyping topic are trying to focus our attention on the geterogensity of asthma in general. By presenting our two cases we would like put a stress on the variety features inside the group of patients with asthma associated with obesity or overweight. This study describes two clinical cases with uncontrolled asthma. Both patients received inhaled therapy with fixed combination IGCS / LABA (1000 mg by fluticasone / salmeterol).

## Method

Clinical case 1: male,27 year-old, with early start of asthma- at the age of 6. The main complaints were shortness of breath, predominantly daytime symptoms, the requirement of rescue medication despite the control medication 4-5 times a week; history of 1 exacerbation in the past year. Comorbidities: Allergic rhinitis under control. BMI 31.4%, ACQ 2.43. According to blood count - neutrophils 72.7%, absolute value 7.8x10^9, eosinophils 0.032. FEV1 57.6%. Sputum analysis – less than 3 neutrophils, 4 eos.

Clinical case 2: female,57 year-old, late asthma onset at age of 48 during the menopause period. The main complaints were cough, night symptoms, shortness of breath on exertion, daily use of rescue medication. No atopic anamnesis; history of 2 exacerbation in the current year. BMI 22%, WC 91 cm. According to blood count - neutrophils 65.6%, absolute value 5.5x10^9, eosinophils 1.6%, absolute value 0.013. Sputum analysis – 4 neutrophils, 2 eos. FEV1 64%.

The period of supervision was 12 month for both cases.

## Results

Type of therapy: Clinical case1: lifestyle and behavior correction. Pharmacotherapy was not changed. Outcome: controlled asthma due to clinical and functional parameters in parallel BMI reduction- BMI 26.6% (BMI initial increase on the first controlled time point could be the result of redistribution of fat and muscle tissue’s mass.

Clinical case 2: Type of therapy IGCS dosage was not escalated, additional control medication was prescribed (antileukotrienes) Outcome partly controlled. Positive inflammation status observed in both cases.

## Conclusion

The asthma control level assessment should be complex and include inflammation parameters, weight status (BMI, WC) in oder to develop individual schemes in therapy algorithm in overweight patients.

## Consent

Written informed consent was obtained from the patient for publication of this abstract and any accompanying images. A copy of the written consent is available for review by the Editor of this journal.

